# Assessment of Use and Safety of Edaravone for Amyotrophic Lateral Sclerosis in the Veterans Affairs Health Care System

**DOI:** 10.1001/jamanetworkopen.2020.14645

**Published:** 2020-10-05

**Authors:** Michelle Vu, Kathryn Tortorice, Jennifer Zacher, Diane Dong, Kwan Hur, Rongping Zhang, Chester B. Good, Peter A. Glassman, Francesca E. Cunningham

**Affiliations:** 1 Pharmacy Benefits Management Services, Center for Medication Safety, Department of Veterans Affairs, Hines, Illinois; 2 Center for Health Equity Research and Promotion, Department of Veterans Affairs, Pittsburgh, Pennsylvania; 3 Pharmacy Benefits Management Services, Department of Veterans Affairs, Hines, Illinois; 4 Division of Insurance, UPMC Health Plan, Pittsburgh, Pennsylvania; 5 Greater Los Angeles Healthcare System, Department of Veterans Affairs, Los Angeles, California; 6 Pharmacy Benefits Management Services, Department of Veterans Affairs, Washington, DC

## Abstract

**Importance:**

Using real-world data, the US Department of Veterans Affairs (VA) initiated a surveillance evaluation of edaravone after its approval for amyotrophic lateral sclerosis (ALS) in 2017. The use and safety of edaravone for patients with ALS in the VA health care system remain to be assessed.

**Objective:**

To describe a pharmacovigilance surveillance initiative with edaravone to monitor patient characteristics, utilization (edaravone cycles and riluzole use), and safety and to evaluate safety/effectiveness.

**Design, Setting, and Participants:**

This propensity score–matched cohort study used data on 369 patients with documented definite or probable ALS in the Veterans Health Administration (VHA) with at least 1 prescription for edaravone between August 1, 2017, and September 30, 2019. The analysis compared edaravone (alone or with riluzole) with riluzole only. For chronic users (≥6 months of drug), a time-to-event model evaluated ALS-related outcomes, with censoring at outcome, death, or end of evaluation. Patients with Parkinson disease, dementia, schizophrenia, or significant respiratory insufficiency per diagnosis codes within 2 years before prescription initiation were excluded. In overall matched cohorts, 223 patients treated with edaravone were 1:3 propensity score matched based on predefined confounders. For the chronic user subgroup analysis, 96 patients receiving edaravone and 424 patients receiving riluzole only were included.

**Exposures:**

Edaravone (alone or with riluzole) vs riluzole only.

**Main Outcomes and Measures:**

Patient characteristics, ALS drug use, and mortality. Acute outcomes (within 6 months of index) included proportion and mean time to event for death, discontinuation, or all-cause hospitalization, and outcomes for chronic users (receiving >6 months of treatment) included hazard ratios of outcomes related to disease-state progression.

**Results:**

Of 369 patients who received edaravone, most were older (mean [SD] age, 64.6 [11.3] years), male (346 [93.8%]), and White (261 [70.7%]). As of September 2019, 59.9% of edaravone patients had discontinued treatment; of those, 49.5% (108 of 218) received only 1 to 3 treatment cycles. Approximately 30% (110 patients) died. In a matched evaluation, significantly more acute all-cause hospitalization events occurred with edaravone (35.4% vs 22.0% for riluzole only); 72.6% of the edaravone cohort received edaravone with riluzole. Among chronic users, edaravone patients (70.8% edaravone with riluzole) had an increased hazard ratio of ALS-associated hospitalization (2.51; 95% CI, 1.18-8.16). The death rate was lower with edaravone but the difference was not statistically significant.

**Conclusions and Relevance:**

Early edaravone discontinuation was common in the VA. Although outcomes favored use of riluzole only in the matched analysis, results should be interpreted with caution, as unmeasured bias in observational data is likely.

## Key Points

**Question:**

What is the real-world experience with edaravone in patients with amyotrophic lateral sclerosis (ALS) within a national integrated health care system?

**Findings:**

In this cohort study of data from 369 US veterans with documented or probable ALS, a significantly greater proportion of acute all-cause hospitalizations was associated with edaravone treatment and, among users receiving at least 6 months of treatment, an increased likelihood of ALS-related hospitalization was associated with edaravone treatment compared with riluzole-only treatment.

**Meaning:**

While these findings should be interpreted with caution, in this evaluation, edaravone (mostly used with riluzole) was associated with more hospitalizations compared with riluzole-only therapy; more evidence is needed to evaluate edaravone treatment outcomes in real-world settings.

## Teaser

This cohort study uses real-world data to assess patient characteristics and use as well as the safety and effectiveness of edaravone among patients with amyotrophic lateral sclerosis in the US Department of Veterans Affairs Health Care System.

## Introduction

Since 2005, the US Department of Veterans Affairs (VA) health care system has provided care for more than 4000 veteran patients with amyotrophic lateral sclerosis (ALS), one of the largest ALS patient populations in a US health care system. Within the Veterans Health Administration (VHA), ALS is a presumptive service connection (eg, subsequent ALS diagnosis is related to veteran’s prior military service), with automatic qualification for VA benefits, including prescription coverage.^1^ Pharmacotherapy has been limited, with only 2 treatments approved for ALS by the US Food and Drug Administration (FDA).^2,3^ Edaravone was approved by the FDA in May 2017.^4^ Prior to edaravone approval, riluzole was the only FDA-approved treatment.^5^ Riluzole was shown to significantly increase survival at 12 and 21 months in a randomized placebo-controlled trial; however, median survival was improved by only approximately 100 days (532 days for riluzole; 449 days for placebo).^6^ Moreover, this benefit was not observed in patients aged 75 years and older or those with advanced ALS.^7^ Survival was not assessed in the edaravone clinical trial.^8^

Edaravone was approved based on a clinical trial in Japanese patients demonstrating some benefit in delaying ALS progression, with inclusion criteria based on a post hoc subgroup analysis.^8^ These criteria limited treatment to patients with early-stage ALS (<2 years from diagnosis) who were independent and well functioning (ALS Functional Rating Scale–Revised [ALS-FRS-R] score ≥2 per item [12 items, each rated on a 5-point scale, 0 indicating inability to perform and 4 indicating normal ability], forced vital capacity ≥80%). Notably, less than 7% of ALS patients meet these stringent inclusion criteria.^9,10,11^ Some observational studies on edaravone use, safety, and effectiveness include non-US, nonveteran populations.^10,11,12,13^

Given limited real-world data on edaravone and the number of veteran patients with ALS, the VA Pharmacy Benefits Management (PBM) designated edaravone for prior authorization drug review nationally, to ensure consistent adjudication, track use, and support appropriate use. The VA Center for Medication Safety (VAMedSAFE) developed a surveillance effort within a national, integrated system and sought to answer the question of how to leverage these real-world data to monitor patient characteristics and use, as well as assess the safety and effectiveness of edaravone.

## Methods

This VAMedSAFE surveillance initiative and its assessment were reported according to the Revised Standards for Quality Improvement Reporting Excellence (SQUIRE 2.0) guidelines. This database drug-use evaluation received approval from the VA institutional review board. A waiver of informed consent was granted because the database research involved no more than minimal risk to the patients reviewed and therefore could not be practicably carried out without the requested waiver of alteration per VA policy.

### Program Description/Surveillance Methods

#### PBM National Prior Authorization Drug Review Program

The VA PBM finalized Criteria for Use (CFU) for edaravone in July 2017, based on evidence from clinical trials and input from subject matter experts; by contrast, riluzole does not have CFU in the VA.^14^ The edaravone CFU inclusion criteria allow its use in patients with preserved ability to walk or self-feed, providing access to treatment for a much broader ALS population compared with clinical trial criteria^8^ or non-VA health plans.^15^ Selected exclusion criteria include significant respiratory insufficiency or difficulty as measured by ALS-FRS-R subscores and comorbidities that complicate assessment of ALS (eg, Parkinson disease, dementia, and schizophrenia).^14^ The ALS-FRS-R is an assessment scale for functional impairment, with cumulative scores from 12 items ranging from 0 indicating worst to 48 indicating best, and was used in edaravone clinical trials.^16,17^

For prior authorization drug review, an ALS-FRS-R score (that reflects function at the time of the request) and other documentation are submitted via an interfacility consultation to a central data set, where information is reviewed and, when indicated, approved centrally by a pharmacy team. While the CFU includes discontinuation criteria and recommendations for monitoring respiratory function and assessing ALS-FRS-R score every 6 months,^14^ decisions to continue therapy and follow-up are deferred to local facilities and professionals.

#### VAMedSAFE Surveillance Initiative

VAMedSAFE conducts biannual descriptive assessments of edaravone use and safety events among the cohort of patients who have received 1 or more prescriptions of edaravone within the VHA. The current cohort assessment includes data from August 1, 2017, to September 30, 2019 (n = 369). Per the CFU, all eligible patients must have documented definite or probable ALS, according to El Escorial–revised Airlie House criteria.^14^ Clinical data were collected until edaravone discontinuation or death, but all patients were assessed as part of the overall cohort.

### Data Sources

Prescription data on edaravone and riluzole were obtained from VA PBM databases and Corporate Data Warehouse. Diagnostic, procedure, and demographic data were retrieved from the National Patient Care Database and Corporate Data Warehouse. Date of death was identified from the Vital Status file.^18^ The ALS-FRS-R score at the time of consultation was collected from the national prior authorization drug review database, where available. Edaravone adverse drug reactions (ADRs) reported by clinicians were obtained from the VA Adverse Drug Event Reporting System (VA-ADERS) database.^19^

### Patient Baseline Characteristics

Patient demographic characteristics included age, sex, race/ethnicity, and marital status (Table 1). Comorbidities per CFU exclusion criteria (eg, Parkinson disease, dementia, and schizophrenia) were identified using *International Classification of Diseases, Ninth Revision (ICD-9)* or *International Statistical Classification of Diseases and Related Health Problems, Tenth Revision (ICD-10)* codes from inpatient or outpatient data. Veteran priority status and prescription benefits were assessed and included to confirm patient access to VA health care. Enrollment was assessed for fiscal year 2017 (October 1, 2016–September 30, 2017; first fiscal year of edaravone availability in the VHA) or later. The ALS-FRS-R score on the consultation was summarized.

**Table 1.** Baseline Characteristics of 369 Patients With at Least 1 Edaravone Prescription

Characteristic	Edaravone users, No. (%)
Age at initiation, mean (SD), y	64.6 (11.3)
Male sex	346 (93.8)
White race^a^	261 (70.7)
Married^a^	275 (74.5)
Exclusion comorbidity^b^	23 (6.2)
VA priority rating, service connection	
50%-100%	356 (96.5)
<50%	3 (0.8)
VA copay	
No copay	356 (96.5)
Prescription copay only	7 (1.9)
Enrollment after fiscal year 2017	123 (33.3)
ALS-FRS-R score on consultation, median (IQR)^c^	36 (31-41)

Abbreviations: ALS-FRS-R, Amyotrophic Lateral Sclerosis Functional Rating Scale–Revised; IQR, interquartile range; VA, US Department of Veterans Affairs.

^a^ Race, marital status missing for 7 patients, demographic information updated as of November 2019.

^b^ Presence of diagnosis code for Parkinson disease, dementia, and/or schizophrenia.

^c^ ALS-FRS-R includes 12 questions, each of which is rated on a 5-point scale, with 0 indicating inability to perform and 4 indicating normal ability, for a cumulative reported score ranging from 0 indicating worst to 48 indicating best. Missing prior authorization drug review consultation score for 12 patients.

### Descriptive Measures

Descriptors included drug use and safety or mortality. Measures included treatment cycles, discontinuation rates, and riluzole use.^20^ CFU criteria require that edaravone be administered in accordance with the package insert.^14^ For maintenance treatment, edaravone is administered as an intravenous (IV) infusion daily for 10 of 14 days, followed by a 14-day drug-free period (28-day cycle).^4^ Given that some cycles were administered by community care professionals and likely not captured in VA data, cycles were estimated using the days from the first to last edaravone prescription fill, divided by 28. Discontinuation was assumed if no further treatment cycles were recorded within 60 days of the last dose and before the end of the evaluation period. Patients receiving edaravone included those with riluzole use (edaravone with riluzole), defined as receiving 1 or more riluzole prescription fills after initiation of and before discontinuation of edaravone treatment, and those without riluzole use (edaravone alone). Riluzole use prior to edaravone initiation was also categorized.

Measures of mortality and voluntarily reported edaravone ADRs were described. Proportion of deaths and time from edaravone initiation until death were summarized for the cohort of edaravone ever-users.^11^ Edaravone-related ADRs reported to VA-ADERS during the surveillance period were described by severity, ADR type, and frequency.

### Comprehensive Safety and Effectiveness Evaluation

VAMedSAFE conducted a retrospective, propensity score–matched cohort evaluation comparing the safety and effectiveness of edaravone use (alone or with riluzole) with riluzole only. Data sources and variables were the same as described in Surveillance Methods, adding inpatient hospitalizations and ALS-FRS-R scores from the Corporate Data Warehouse that were manually entered into the electronic health record prespecified fields (ie, data in free text were not captured). The cohorts consisted of veteran patients with a diagnosis of ALS and 1 or more prescription fills of edaravone or riluzole between August 1, 2017, and December 31, 2018 (eFigure in the Supplement). Exclusion criteria reflect CFU exclusion and discontinuation criteria^14^ and included those with diagnosis codes for excluded comorbidities, dyspnea, or orthopnea, or procedure codes for bilevel positive airway pressure, mechanical ventilation, or tracheostomy within 2 years before prescription initiation.

Edaravone and riluzole-only users were matched 1:3, using a caliper width of 0.2 of the logit of the propensity score.^21^ The propensity score was developed using predefined baseline confounders of receipt of edaravone and ALS progression (age ≥65 years, sex, race/ethnicity, marital status, priority group, VA copay, and duration of ALS).^11,12,13^ Duration of ALS was categorized as less than or equal to 2 years, more than 2 years, or unknown,^8^ based on the first date of a documented ALS diagnosis code to date of edaravone initiation. If the first ALS diagnosis code occurred after edaravone initiation, the duration of ALS was categorized as unknown. Balance after matching was assessed by a standardized mean difference of less than 0.1.^21^

The index date was the first prescription fill of edaravone or riluzole within the period August 1, 2017, to December 31, 2018. Acute outcomes, defined as events occurring within the 6-month follow-up period after the index date,^10^ were assessed for the overall matched cohorts. These included proportions of deaths, discontinuations (no treatment cycle received/prescription possession of riluzole within 60 days after the last dose and before the end of follow-up), or all-cause hospitalizations. Mean time to event and change in ALS-FRS-R score from baseline (closest score to the index date) to end of follow-up (closest score to the 6-month follow-up date) were also assessed.

### Statistical Analysis

An analysis was conducted for chronic users of each cohort, defined as those receiving at least 6 months of treatment.^11,13^ As in the overall analysis, death was included. Additional outcomes were evaluated to assess ALS progression. These included hospitalization associated with ALS, dyspnea, or orthopnea (per diagnosis codes) and surrogate markers of functional decline (procedure codes for tracheostomy, mechanical ventilation, and percutaneous endoscopic gastrostomy [PEG] tube placement), reflecting CFU discontinuation criteria.^14^ Cox proportional hazards regression models were used to estimate hazard ratios (HRs) with 95% CIs for chronic outcomes. Veteran patients were followed from the index date to the date of the first outcome, prescription discontinuation, death, or the end of evaluation, whichever came first. Additionally, frequency of events, mean time to event, and events per 100 person-years of follow-up were summarized. Analysis was conducted using SAS version 9.2 (SAS Institute Inc).

## Results

### Patient Baseline Characteristics

As of September 2019, 369 veteran patients had received edaravone through the VHA. At baseline, the mean (SD) age was 64.6 (11.3) years and 346 were male (93.8%), 261 White (70.7%), and 356 fully covered for prescription drugs (96.5%) (Table 1). Approximately one-third were enrolled in VA care after fiscal year 2017; these patients received their first dose of edaravone a mean (SD) of 197 (182.6) days after the date of enrollment. The median ALS-FRS-R score documented prior to edaravone initiation was 36 (interquartile range [IQR], 31-41).

### Descriptive Measures

The estimated number of edaravone cycles per patient showed substantial variation (mean [SD] number of cycles, 8.7 [7.4]). Of patients who received at least 1 cycle, 40.1% (151 of 369) continued to receive edaravone until September 2019 (Table 2). The median number of edaravone cycles for patients who discontinued treatment was 4 (IQR, 1-9) (eg, 49.5% [108 of 218] received 1 to 3 treatment cycles). The ALS-FRS-R score on consultation was similar between those who continued (median, 35; IQR, 31-40) and discontinued treatment (median, 37; IQR, 32-41.5). Many patients (71.3% [263 of 369]) received edaravone with riluzole (eTable 1 in the Supplement). For patients with riluzole use prior to edaravone (255 of 369), many (92.5% [236 of 255]) continued use after initiating edaravone treatment (eTable 2 in the Supplement).

**Table 2.** Edaravone Use and Baseline Score of Veteran Patients, Stratified by Discontinuation

Variable	Edaravone users, No. (%)		
	Overall	Treatment	
		Discontinued^a^	Continued
Total patients	369	218 (59.9)	151 (40.1)
Cycles, median (IQR)^b^	7 (2-14)	4 (1-9)	13 (6-21)
Cumulative cycles, No. (column %)			
1	85 (23.0)	73 (33.5)	12 (7.9)
2	20 (5.4)	12 (5.5)	8 (5.3)
3	27 (7.3)	23 (10.6)	4 (2.6)
4	15 (4.1)	11 (5.0)	4 (2.6)
5	19 (5.1)	12 (5.5)	7 (4.6)
≥6	203 (55.0)	87 (39.9)	116 (76.8)
ALS-FRS-R score on consultation, median (IQR)^c^	36 (31-41)	35 (31-40)	37 (32-41.5)

Abbreviations: ALS, amyotrophic lateral sclerosis; ALS-FRS-R, Amyotrophic Lateral Sclerosis Functional Rating Scale–Revised; IQR, interquartile range.

^a^ Defined as no treatment cycles were received within 60 days after the last dose and before the end of prescription data (September 30, 2019).

^b^ Estimated based on first and last prescription fills in the VA system.

^c^ ALS-FRS-R includes 12 questions, each of which is rated on a 5-point scale, with 0 indicating inability to perform and 4 indicating normal ability, for a cumulative reported score ranging from 0 indicating worst to 48 indicating best. Missing prior authorization drug review consultation score for 12 patients.

Of the 369 patients, 110 died (29.8%); a mean (SD) of 260.3 (170.5) days elapsed from their first edaravone prescription to the date of death. Twelve ADRs were reported to VA-ADERS: 3 mild, 5 moderate, and 4 severe. For mild or moderate ADRs, types of ADRs included IV port thrombosis, product bag leakage, dyspnea or chest discomfort, anemia, chills or tremor, and rash. Among severe edaravone ADRs reported (eg, ADRs associated with hospitalization, urgent intervention, and/or risk of organ damage), there was 1 death, associated with progression of liver disease in a patient with liver disease prior to edaravone initiation. The other 3 severe ADR reports described dyspnea or leg edema, pneumonia and pulmonary embolism, and pneumonia or respiratory depression.

### Comprehensive Safety and Effectiveness Evaluation

For the propensity score–matched analysis, 223 patients receiving edaravone (72.6% edaravone with riluzole, 27.4% edaravone alone) (eTable 1 in the Supplement) were matched to 669 patients receiving riluzole-only treatment (eFigure in the Supplement). Propensity score–matching diagnostics showed balance between edaravone and riluzole-only groups (eTable 3 in the Supplement). The overall matched groups were mostly diagnosed with ALS in the past 2 years (68.6% edaravone; 68.5% riluzole) (Table 3). The ALS-FRS-R scores were not well populated in VA databases; where available, baseline scores were similar. Chronic users consisted of 43% of patients receiving edaravone (n = 96) and 63% of patients receiving riluzole-only treatment (n = 424), and their baseline characteristics were similar. Of edaravone chronic users, 70.8% received edaravone with riluzole (eTable 1 in the Supplement).

**Table 3.** Comprehensive Safety and Effectiveness Evaluation: Select Baseline Characteristics for Matched Patients

Variable	No. (%)			
	Overall matched cohorts^a^		Chronic users^b^	
	Edaravone (n = 223)	Riluzole only (n = 669)	Edaravone (n = 96)	Riluzole only (n = 424)
Age ≥65 y	130 (58.3)	389 (58.2)	54 (56.3)	229 (54.0)
Male	216 (96.9)	649 (97.0)	95 (99.0)	410 (96.7)
White race	164 (73.5)	489 (73.1)	81 (84.4)	322 (75.9)
Duration of ALS, y^c^				
≤2	153 (68.6)	458 (68.5)	66 (68.8)	296 (69.8)
>2	20 (9.0)	62 (9.3)	12 (12.5)	45 (10.6)
Unknown	50 (22.4)	149 (22.3)	18 (18.8)	83 (19.6)
ALS-FRS-R score, median (IQR)^d^	38 (37-42)	35 (30-39)	39.5 (30-42)	35 (31-38)

Abbreviations: ALS, amyotrophic lateral sclerosis; ALS-FRS-R, Amyotrophic Lateral Sclerosis Functional Rating Scale–Revised; IQR, interquartile range; VA, US Department of Veterans Affairs.

^a^ Propensity score matched on age 65 years or older, sex, race, marital status, priority group, VA copay, and duration of ALS.

^b^ Receiving at least 6 months of treatment.

^c^ Based on the first date an ALS diagnosis code was listed in an encounter to the date of edaravone initiation. If the first ALS diagnosis code occurred after edaravone initiation, the duration of ALS was categorized as unknown.

^d^ Available for 215 edaravone (from consultation) and 26 riluzole patients (from VA Corporate Data Warehouse) for overall and 95 edaravone (from consultation) and 17 patients who received riluzole (from VA Corporate Data Warehouse) for chronic users.

Acute outcomes were summarized for the overall matched cohorts (Table 4). Death rates were similar between cohorts. There was a significantly greater proportion of acute all-cause hospitalization events in the edaravone cohort (35.4% edaravone; 22.0% riluzole-only; Bonferroni-corrected *P* < .001). Moreover, time to hospitalization was shorter within the edaravone cohort (mean [SD], 44.5 [47.7] days for edaravone; 68.2 [53.2] days for riluzone-only treatment; *P* = .001). Time to treatment discontinuation was significantly shorter for edaravone (mean [SD], 93.8 [81.7] days for edaravone; 161.8 [113.7] days for riluzole only; *P* < .001). Decrease from baseline ALS-FRS-R score was greater in the riluzole-only cohort (median, −8; IQR, −14 to −4 for riluzole-only vs −2; IQR, −6 to −1 for edaravone).

**Table 4.** Comprehensive Safety and Effectiveness Evaluation: Acute Outcomes (Within 6 Months of Drug Initiation) for Matched Cohorts

Variable	No. (%)		*P* value^a^
	Edaravone (n = 223)	Riluzole-only (n = 669)	
Events overall	122 (54.7)	340 (50.8)	.35
Death	25 (11.2)	101 (15.1)	.18
Discontinuation^b^	64 (28.7)	227 (33.9)	.16
Hospitalization (all-cause)	79 (35.4)	147 (22.0)	<.001^c^
Time to event, mean (SD), d			
Death	76.3 (48.0)	99.1 (54.0)	.06
Discontinuation^b^	93.8 (81.7)	161.8 (113.7)	<.001^c^
Hospitalization (all-cause)	44.5 (47.7)	68.2 (53.2)	.001^c^
Change in ALS-FRS-R score, median (IQR)^d^	−2 (−6 to −1)	−8 (−14 to −4)	

Abbreviations: ALS, amyotrophic lateral sclerosis; ALS-FRS-R, Amyotrophic Lateral Sclerosis Functional Rating Scale–Revised; IQR, interquartile range.

^a^ Bonferroni-corrected significance is at *P* = .017.

^b^ Defined as no treatment cycles were received within 60 days before the end of follow-up (June 30, 2019).

^c^ Bonferroni corrected.

^d^ Available for 9 edaravone and 26 riluzole patients.

Among chronic users, the riluzole-only subcohort (n = 424) had longer time to follow-up (thus drug exposure) compared with edaravone (n = 96) (mean [SD], 403.9 [113.6] days for riluzole-only; 305.1 [77.8] days for edaravone). The discontinuation rate was also higher with riluzole-only treatment vs edaravone (19.3% vs 11.5%). Hazard ratios of hospitalization associated with ALS progression (HR, 2.51; 95% CI, 1.18-8.16) and PEG tube placement (HR, 3.04; 95% CI, 1.25-10.66) were higher with edaravone compared with riluzole-only treatment (Table 5). There were few (<10%) incident mechanical ventilation and tracheostomy events. Decreases in ALS-FRS-R score from baseline (available for 4 edaravone; and 17 patients who received riluzole only) were greater within the riluzole-only subcohort (median, −8; IQR, −14 to −6 for riluzole-only; −3.5; IQR, −8 to 0.5 for edaravone). In addition, there was no statistically significant difference between subgroups, although death rates (per 100 patient-years) were lower for edaravone (29.3 riluzole-only; 18.0 edaravone; HR, 0.77; 95% CI, 0.43-1.18).

**Table 5.** Outcomes of Chronic User Subcohorts^a^

Outcome	Cohort (No.)	Events, No. (%)	Time to event, mean (SD), d	Follow-up, person-years	Events per 100 person-years	HR (95% CI)^b^
Death	Edaravone (96)	22 (22.9)	340.4 (80.8)	122.3	18.0	0.77 (0.43-1.18)
	Riluzole-only (424)	182 (42.9)	475.5 (201.4)	620.9	29.3	
Hospitalization (ALS, dyspnea, or orthopnea)	Edaravone (96)	18 (18.8)	254.4 (174.7)	111.5	16.1	2.51 (1.18-8.16)
	Riluzole-only (424)	35 (8.3)	199.2 (153.0)	588.6	5.9	
Tracheostomy	Edaravone (96)	4 (4.2)	395.8 (99.4)	120.8	3.3	1.22 (0.29-1.63)
	Riluzole-only (424)	16 (3.8)	283.7 (160.3)	607.8	2.6	
Mechanical ventilation	Edaravone (96)	9 (9.4)	320.1 (167.2)	118.1	7.6	2.63 (0.90-6.45)
	Riluzole-only (424)	17 (4.0)	279.4 (165.4)	607.9	2.8	
PEG tube placement	Edaravone (96)	14 (14.6)	255.9 (167.0)	112.8	12.4	3.04 (1.25-10.66)
	Riluzole-only (424)	22 (5.2)	205.2 (137.7)	601.5	3.7	

Abbreviations: ALS, amyotrophic lateral sclerosis; HR, hazard ratio; PEG, percutaneous endoscopic gastrostomy.

^a^ Receiving at least 6 months of treatment.

^b^ Comparing hazard ratios of the edaravone group to riluzole-only subgroups and Bonferroni-corrected confidence intervals.

## Discussion

To our knowledge, this evaluation is the largest pharmacovigilance initiative of edaravone users in a US health care system, providing a real-world analysis of patient characteristics, use, safety, and effectiveness of edaravone use within the VHA. As of September 2019, 369 veteran patients, with relatively high ALS-FRS-R scores at initiation, had received edaravone. The VA provides an important point of access to this treatment option, given that most edaravone users had no prescription copay (97%) and 33% enrolled in VA care in fiscal year 2017 or later. In this descriptive monitoring of use, edaravone discontinuation was common (approximately 60%). Additionally, in those who discontinued, a clinically significant proportion (49.5%) received only 1 to 3 infusions.

When comparing use of edaravone (alone or with riluzole) with riluzole-only use, we found that significantly greater acute all-cause hospitalization events were associated with edaravone compared with riluzole-only use. Among chronic users, edaravone was similarly associated with increased hazard ratios of ALS-associated hospitalization. Notably, although times to PEG placement, tracheostomy, and mechanical ventilation were longer for edaravone chronic users compared with riluzole-only, edaravone was significantly associated with increased hazard ratios for PEG placement and not significantly associated with tracheostomy and mechanical ventilation benefits. Death was less common among edaravone chronic users (18.0 per 100 patient-years; 29.3 per 100 patient-years riluzole-only treatment); however, this finding was not statistically significant (HR, 0.77; 95% CI, 0.43-1.18). Overall, the comprehensive evaluation findings may suggest mixed results with disease-progression and safety events as well as increased hazard ratios of ALS-associated hospitalization with edaravone use relative to riluzole-only use.

### Strengths and Limitations

The strengths of this comprehensive surveillance initiative are its large size and its comparative evaluation. Our measures for use, safety, and effectiveness are consistent with those reported from other health systems.^10,11,12,20^ Additionally, database methods allowed for timely national surveillance compared with retrospective medical record review.^10^ In the propensity-matched cohort evaluation, the edaravone cohort included many patients who used edaravone with riluzole (72.6%), which reflects the real-world dual use of these treatments. Notably, the edaravone clinical trial allowed patients to continue receiving riluzole, and its use was common (91%).^18^

These results supplement the only other published US evaluation of edaravone use and safety by Jackson et al.^20^ By 1 year after approval, 3007 patients received edaravone, with 1006 discontinuing use. The authors surveyed 75 physician prescribers of edaravone, and based on 40 respondents, 67% of edaravone patients used riluzole concurrently and 43% opted for home-infusion treatment. Additionally, from the manufacturer’s safety database, 817 ADRs were reported. There were 272 severe ADRs, including death (104 of 272), dyspnea (19 of 272), and pneumonia (17 of 272).^20^ Overall, these postmarketing ADRs were similar to those reported to VA-ADERS and may reflect ALS progression. Exceptions noted were pulmonary embolism (6 severe reports in manufacturer database; 1 in VA-ADERS) and injection-related complications (9 severe injection-site infections in manufacturer database; 2 portal vein thrombosis events at infusion in VA-ADERS). VAMedSAFE will continue to conduct biannual surveillance of these and other safety signals.

To our knowledge, this is the first evaluation within a US health care system to use a matched-cohort, time-to-event analysis to assess safety and effectiveness of edaravone compared with an active comparator. Okada et al^13^ used survival analysis to compare tracheostomy-free survival between Japanese edaravone users (n = 27) and a historical control cohort (n = 30), for up to 80 months; a survival benefit was associated with edaravone treatment (HR for death, 0.37; 95% CI, 0.20-0.74). However, 37% (10 of 27) discontinued edaravone but were not censored, meaning survival was not associated with drug exposure. Within an Italian ALS clinic, edaravone users (n = 31) were compared with a historical-control cohort (n = 50); findings suggested no clinically or statistically significant differences in ALS-FRS-R score, respiratory function, or muscle strength, at 3- or 6-month follow-up.^12^ Abraham et al^11^ compared edaravone users with at least 6 months of treatment (n = 20) with contemporaneous non-edaravone users (71) at 1 Israeli clinic. There was no difference in effectiveness, but mortality was numerically greater in the edaravone group. None of these studies used matching, and the latter 2 studies^11,12^ did not account for differential follow-up or censoring due to death or tracheostomy.

This evaluation has limitations. First, the edaravone cohort was overall less healthy^14^ than edaravone clinical trial participants,^8^ which may explain the disease progression observed. Importantly, because riluzole is not regulated by VA CFU and is an oral drug whereas edaravone requires infusions, riluzole-only users are likely different from edaravone users in complex ways. Potentially, some riluzole users were sicker; edaravone is reserved for ALS patients with good functional status per CFU. Alternatively, edaravone patients may include those who progressed while receiving riluzole, and hence were different from those continuing to receive riluzole only. Consequently, despite our efforts to match patients receiving edaravone and those receiving riluzole-only treatment, residual confounding or baseline differences are likely. To maintain a larger pool of matching candidates, we did not use a new-user design for the riluzole-only cohort, which introduces bias toward favorable responders to riluzole. Matching on additional factors may improve comparison, including ALS-FRS-R score components, ALS-onset type (bulbar vs nonbulbar), and pulmonary and muscular function.^10^ Second, some outcomes were infrequently captured in VA databases (eg, ALS-FRS-R score and functional-status surrogate markers). Edaravone exposure was based on only the first and last prescription fill dates within the VHA, as we could not capture cycles received in non-VHA settings. In addition, we did not exclude PEG-tube use at baseline (allowed per CFU) and could not assess if PEG-tube outcomes were incident (reflecting decreases in function or discontinuation criterion).

Overall, the challenge of assessing edaravone safety and effectiveness is that ALS progression is unpredictable and heterogeneous.^3,10,11,13^ Simply, are adverse events related to disease progression, treatment, or both? Disease complications, death, or treatment discontinuation may occur prior to evaluation of drug effectiveness. In our evaluation, patients receiving riluzole-only treatment continued to receive treatment longer (mean 403.9 days, riluzole-only treatment; mean 305.1 days, edaravone), given differential censoring. This censoring bias may be addressed in future studies (if effectiveness is the primary interest) by applying survival models accounting for competing risks^22^ (eg, accounting for differential death or discontinuation between comparators).

## Conclusions

The results of this study should be construed as exploratory given inherent methodologic limitations. Notably, this surveillance evaluation identified that patients using edaravone (mostly with riluzole) had unexpected outcomes, which raises questions about its benefit in VHA patients and underscores the importance of further studies. To confirm these findings, robust research designs with in-depth medical record review are needed to better characterize drug exposure, reasons for discontinuation, outcomes, and covariates. The VA will continue to track and monitor the safe and appropriate use of edaravone and provide timely information to optimize veteran ALS care.

## Notes

Supplement. **eFigure.** Flowchart of Descriptive Assessment, Matched Cohort Design, and Chronic User Sub-Analysis **eTable 1.** Riluzole Use Patterns Among Edaravone Patients **eTable 2.** Riluzole Use Continuation Among Patients With Riluzole Use Prior to Edaravone Initiation **eTable 3.** Comprehensive Safety and Effectiveness Evaluation-Matched Edaravone and Riluzole-Only Users
