# Supplementary material for: Assessment of Use and Safety of Edaravone for Amyotrophic Lateral Sclerosis in the Veterans Affairs Health Care System
Source: JAMA Netw Open. 2020 Oct 5;3(10):e2014645. doi: 10.1001/jamanetworkopen.2020.14645 (PMC7536587; doi:10.1001/jamanetworkopen.2020.14645)
Supplement: Supplement. — eFigure. Flowchart of Descriptive Assessment, Matched Cohort Design, and Chronic User Sub-Analysis eTable 1. Riluzole Use Patterns Among Edaravone Patients eTable 2. Riluzole Use Continuation Among Patients With Riluzole Use Prior to Edaravone Initiation eTable 3. Comprehensive Safety and Effectiveness Evaluation-Matched Edaravone and Riluzole-Only Users [file jamanetwopen-e2014645-s001.pdf]

Supplemental Online Content

Vu M, Tortorice K, Zacher J, et al. Assessment of use and safety of edaravone for amyotrophic lateral sclerosis in the Veterans Affairs Health Care System. *JAMA Netw Open*. 2020;3(10):e2014645. doi:10.1001/jamanetworkopen.2020.14645

eFigure. Flowchart of Descriptive Assessment, Matched Cohort Design, and Chronic User Sub-Analysis

eTable 1. Riluzole Use Patterns Among Edaravone Patients

eTable 2. Riluzole Use Continuation Among Patients With Riluzole Use Prior to Edaravone Initiation

eTable 3. Comprehensive Safety and Effectiveness Evaluation-Matched Edaravone and Riluzole-Only Users

This supplemental material has been provided by the authors to give readers additional information about their work.

© 2020 Vu M et al. *JAMA Network Open.*

eFigure. Flowchart of descriptive assessment, matched cohort design, and chronic user sub-analysis

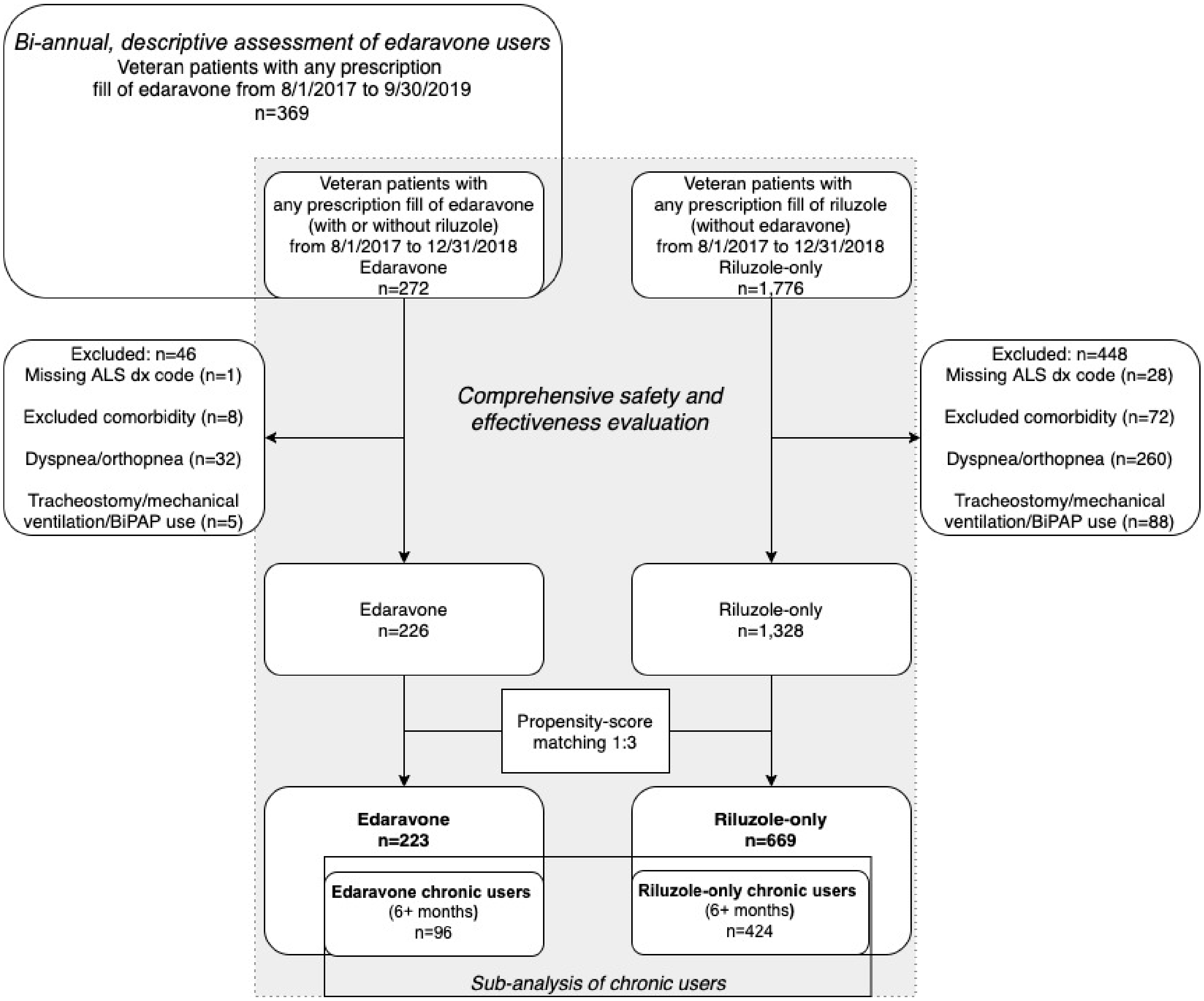

ALS dx: Amyotrophic lateral sclerosis diagnosis code; Excluded comorbidity: Parkinson’s disease, dementia, or schizophrenia; BiPAP: bilevel positive airway pressure

© 2020 Vu M et al. *JAMA Network Open.*

eTable 1. Riluzole use patterns among edaravone patients

	Riluzole use[a]		
	Prior to edaravone initiation[b]	During edaravone treatment period (edaravone with riluzole)[b]	After edaravone discontinuation [b,c]
	n, row %	n, row %	n, row %
**All edaravone users** N=369	255 (69.1%)	263 (71.3%)	134 (36.3%)
**Edaravone matched cohort** N=223	157 (70.4%)	162 (72.6%)	94 (42.2%)
**Edaravone chronic user sub-cohort[d]** N=96	64 (66.7%)	68 (70.8%)	34 (35.4%)

[a] defined as receiving ≥1 riluzole prescription fill during time periods specified, using prescription data from 8/1/2017 to 9/30/2019

[b] time periods are not mutually exclusive (e.g., not all edaravone with riluzole patients had use prior to edaravone initiation)

[c] no further treatment cycles were recorded within 60 days of the last dose and before the end of the evaluation period

[d] receiving ≥6 months of edaravone treatment

eTable 2: Riluzole use continuation among patients with riluzole use prior to edaravone initiation

	Patients with riluzole use prior to edaravone initiation[a] n=	*Riluzole use after edaravone initiation?[a]*	
		Yes (row % of those with prior use):	*No (row % of those with prior use):*
**All edaravone users** N=369	255	*236 (92.5%)*	*19 (7.5%)*
**Edaravone matched cohort** N=223	157	*146 (93.0%)*	*11 (7.0%)*
**Edaravone chronic user sub-cohort[b]** N=96	64	*59 (92.2%)*	*5 (7.8%)*

[a] defined as receiving ≥1 riluzole prescription fill during time periods specified, using prescription data from 8/1/2017 to 9/30/2019.

[b] receiving ≥6 months of edaravone treatment

© 2020 Vu M et al. *JAMA Network Open.*

eTable 3: Comprehensive safety and effectiveness evaluation- matched edaravone and riluzole-only users

	Before matching			After matching		
**Baseline characteristics**	**Edaravone N=226**	**Riluzole-only N=1,328**	**SMD**	**Edaravone N=223**	**Riluzole-only N=669**	**SMD**
	n (%)	n (%)		n (%)	n (%)	
Age ≥65 years	130 (57.5%)	923 (69.5%)	0.25	130 (58.3%)	389 (58.2%)	0
Male	216 (95.6%)	1294 (97.4%)	0.10	216 (96.9%)	649 (97.0%)	0.01
Caucasian	167 (73.9%)	964 (72.6%)	0.03	164 (73.5%)	489 (73.1%)	0.01
Married	172 (76.1%)	947 (71.3%)	0.11	169 (75.8%)	509 (76.1%)	0.01
Priority service connection 50%-100%	224 (99.1%)	1226 (92.3%)	0.34	221 (99.1%)	663 (99.1%)	0
No copay	224 (99.1%)	1227 (92.4%)	0.34	221 (99.1%)	663 (99.1%)	0
Duration of ALS	n (% cat)	n (% cat)		n (% cat)	n (% cat)	
≤2 years	154 (68.1%)	918 (69.1%)	0.02	153 (68.6%)	458 (68.5%)	0.01
>2 years	20 (8.9%)	124 (9.3%)	0.01	20 (9.0%)	62 (9.3%)	0
Unknown	52 (23.0%)	286 (21.5%)	0.04	50 (22.4%)	149 (22.3%)	0.01

SMD=Standardized mean difference

© 2020 Vu M et al. *JAMA Network Open.*
